# Open science and human genetic data: recommendations on South Africa’s *Draft National Open Science Policy*

**DOI:** 10.3389/fgene.2023.1248747

**Published:** 2023-10-02

**Authors:** Donrich Thaldar, Amy Gooden, Michaela Steytler

**Affiliations:** ^1^ School of Law, University of KwaZulu-Natal, Durban, South Africa; ^2^ Petrie-Flom Center for Health Law Policy, Biotechnology, and Bioethics, Harvard Law School, Cambridge, MA, United States

**Keywords:** *Draft National Open Science Policy*, freedom of scientific research, human genetic data, open science, ownership, South Africa

## Abstract

The *Draft National Open Science Policy*, which was shared by the South African government with stakeholders in 2022, is an encouraging step forward as it aims to promote the practice of open science in South Africa through a system of incentives. Since South Africa is constitutionally committed to be an open and democratic society, this approach is preferable to the approach of state control that characterizes the *Draft National Policy on Data and Cloud*—another data-related policy initiative by the South African government. However, there is room for improvement in the *Draft National Open Science Policy*. In particular, it should: (a) rely on the right to freedom of scientific research to strengthen the policy; (b) rectify the omission of ownership from its policy analysis; and (c) retain a clear differentiation between human and non-human genetic data. This will ensure that the final policy is clearly anchored in the South African Constitution, and that the principle of “as open as possible, as closed as necessary” can be applied to human genetic data in a legally well informed and accountable way.

## 1 Introduction

Science is “an indispensable contribution to the human endeavour” (International Science Council, 2020). Science is necessary to advance society, stimulate innovation, enhance education, develop policies, and protect well-being. But science is most successful when knowledge is freely available (International Science Council, 2020). There is growing concern that science has become too secluded to benefit the common good of society (International Science Council, 2020). Therefore, the philosophical concept of *open science* has emerged as an endeavor to close the science–society gap by democratizing scientific knowledge (Britt Holbrook, 2019). Open science aims to empower all to partake in science, aided by the Internet, which allows broad dissemination of knowledge (Bahlai et al., 2019; Heise and Pearce, 2020; Hanwell, 2022). Open science is a “no-barrier approach to scientific research” (Steger and Hantho, 2019) that is based on the principle of the free sharing of scientific knowledge. This entails taking down the barriers, such as article paywalls, that “chronically impede scientific progress” (Crow and Tananbaum, 2020). South Africa’s *Draft National Open Science Policy* (Department of Science and Innovation, 2022) fits well within this philosophical framework.

In this article, we analyze the *Draft National Open Science Policy*, with a focus on a particular kind of scientific knowledge: *human genetic data*. Human genetic data are defined in the *International Declaration on Human Genetic Data* (the *Declaration*) as “information about heritable characteristics of individuals obtained by analysis of nucleic acids or by other scientific analysis” (United Nations Educational Scientific and Cultural Organisation, 2003). As highlighted by the *Declaration*, human genetic data has a special status, because such data (a) can be predictive of genetic predispositions concerning individuals; (b) may have a significant impact on the family, including offspring, extending over generations, and in some instances on the whole group to which the person concerned belongs; (c) may contain information, the significance of which is not necessarily known at the time of collection of the biological samples; and (d) may have cultural significance for persons or groups. Accordingly, the *Declaration* calls for an appropriate level of protection of human genetic data that recognizes the sensitive nature of such data. At the same time, the *Declaration* also recognizes that the use of human genetic data is of “paramount importance” for the progress of life sciences and medicine (United Nations Educational Scientific and Cultural Organisation, 2003).

We identify three aspects of the *Draft National Open Science Policy* that require reconsideration: First, we suggest that the *Draft National Open Science Policy* should have a clear anchoring in the South African Constitution, and that this can best be accomplished by building a conceptual nexus between open science and the right to freedom of scientific research. Second, we suggest that the issue of ownership of human genetic data is important and consequential, and that its omission from the analysis in the *Draft National Open Science Policy* should be rectified. Third, we suggest that a clear differentiation between human and non-human genetic data is justified, and should be retained.

We also highlight a number of positive aspects in the *Draft National Open Science Policy* that are accentuated when it is compared to the *Draft National Policy on Data and Cloud* (Department of Communications and Digital Technologies, 2021)—another draft policy that is relevant to human genetic data, and which was released by the South African government in 2021.

## 2 Analysis

### 2.1 Rely on the right to freedom of scientific research to strengthen the policy

The vision espoused in the *Draft National Open Science Policy* is constituted by the following elements: (a) equality of opportunity; (b) environmental sustainability; (c) democratization of knowledge; (d) inclusive socio-economic development; and (e) scientific research. Given that the South African Constitution is the supreme law of South Africa, and the South African state has a duty to respect, protect, promote, and fulfil the rights in the Bill of Rights, it would significantly strengthen the *Draft National Open Science Policy* if its vision were explicitly grounded in the South African Constitution—in particular its Bill of Rights. This can be achieved by linking the constituent elements of its vision to constitutionally enumerated rights. Such links may be apparent in some instances—such as equality of opportunity and the right to equality—given that the right to equality is well known and often referred to in policy discourse. However, such a link may be less apparent in the case of the element *scientific research*. Does scientific research have any link with the South African Constitution?

The answer is yes. Scientific research enjoys an explicit link with the South African Constitution in the form of the *right to freedom of scientific research* (contained in section 16(1)(*d*)). By invoking the right to freedom of scientific research and unpacking its meaning and purposes, the *Draft National Open Science Policy* can, going forward, provide a more solid basis for the relevance and importance of scientific research—and by extension open science—in South Africa’s constitutional dispensation. The right to freedom of scientific research serves purposes that are at the core of our constitutional value system: promoting individual autonomy, facilitating the search for truth, and supporting democracy (Thaldar and Steytler, 2021). We briefly elaborate on each of these purposes.

#### 2.1.1 Promoting individual autonomy

Freedom of scientific research enables individual scientists to find self-fulfillment in pursuing their calling (Case v Minister of Safety and Security, 1996; Steytler, 2021; Thaldar and Steytler, 2021). While this is in itself valuable (Member of the Executive Council for Education: KwaZulu-Natal v Pillay, 2008; British American Tobacco South Africa (Pty) Ltd v Minister of Health, 2012; Van Breda v Media 24 Ltd, 2017; Jordaan, 2009), it also has a powerful knock-on effect on society—and on the autonomy of individuals in society (Jordaan, 2007). Freedom of scientific research has historically been a catalyst for scientific progress; scientific progress, in turn, has played an important role in improving the human condition (Jordaan, 2007), and has “freed a significant portion of humanity from ignorance, poverty and disease” (Corbellini, 2007). An improved human condition broadens the horizons for individual actualization across society.

#### 2.1.2 Facilitating the search for truth

Science has been described as “the search for truths about the natural world” (Lederberg, 1972). Freedom of scientific research facilitates this search for truth by enabling free research and experimentation, the dissemination of results, and the subjection of methodologies, datasets, and results to scrutiny by other scientists (Steytler, 2021). South Africa’s Constitutional Court—the country’s apex court—has expressed itself in favor of a free marketplace of ideas, based on unfettered supply of, and demand for, ideas (Case v Minister of Safety and Security, 1996).

#### 2.1.3 Supporting democracy

The contemporary understanding of the concept “democracy” is more than just the casting of a vote in an election, and includes values such as transparency, accountability, and participation in public life (Thaldar and Steytler, 2021). Furthermore, South Africa’s Constitutional Court has held that the need for *informed* decision-making has become integral to the contemporary understanding of democracy (South African Broadcasting Corporation Ltd v National Director of Public Prosecutions, 2007). This speaks directly to the importance of freedom of scientific research, as science—when practiced freely—seeks to generate reliable, evidence-based knowledge about the world. Such reliable, evidence-based knowledge about the world enables *informed* decision-making and therefore supports democracy.

Open science, by promoting the transparency and accessibility of knowledge, supports and bolsters the practice of freedom of scientific research. It should also be recognized that freedom of scientific research is a necessary condition for open science. Censorship of the dissemination of the results of scientific research would not only be an infringement of the right to freedom of scientific research, but would also inevitably undermine open science. Clearly, open science is intertwined with freedom of scientific research. We suggest that clearly linking and placing reliance on the constitutional right to freedom of scientific research would add significant legal gravitas to the policy initiative of promoting open science.

### 2.2 Include the issue of ownership in the policy analysis

While the *Draft National Open Science Policy* deals at length with intellectual property rights, it completely omits other kinds of property rights. It is important, for example, to consider not only the copyright in *datasets* of human genetic data, but also common law ownership of the human genetic *data* that make up such datasets. Note that the question of ownership of human genetic data is distinct from—and yet interacts with—data subjects’ privacy rights in their genetic data and researchers’ possible claims to intellectual property rights related to such genetic data. Stated differently, the legal nature of human genetic data is not one-dimensional, but multidimensional (Thaldar et al., 2022). These various legal dimensions interact with each other—one right can limit another in specific, defined ways (Thaldar et al., 2022). It would therefore be a serious mistake to conceptualize human genetic data in only one or two dimensions and ignore the other dimension(s), as this would render an incomplete, and likely incorrect, understanding of the rights applicable to human genetic data. Yet, this is unfortunately what the *Draft National Open Science Policy* does.

We are not alone in calling for policy engagement with the issue of human genetic data ownership. A 2018 report by the Academy of Science of South Africa (ASSAf) entitled *Human Genetics and Genomics in South Africa*: *Ethical, Legal and Social Implications* (ASSAf, 2018) (the ASSAf report) called for this topic to be “carefully and vigorously debated and clarified for the South African context” (ASSAf, 2018). However, the ASSAf report does proffer a substantive position of its own, namely, that the “custodianship” of human genetic data ought to be preferred, and “ownership” avoided (ASSAf, 2018). We suggest that any normative inquiry about the desirability of human genetic data ownership should be informed by *inter alia* existing common law property rights. This is important, not only because respect for existing rights is a well-established norm, but also because the existence of existing rights may pose significant practical legal challenges to policy options that threaten to encroach on existing rights. For example, if, hypothetically, private research company X is the *owner* of the genetic data of thousands of South Africans, a policy that proposes that all genetic data of South Africans ought to be made public property effectively proposes that the state ought to *expropriate* private research company X’s property. This may require an excessive amount of state resources to accomplish, which raises the question of whether the policy objectives (such as greater accessibility of the genetic data) cannot be attained through different means (than making all genetic data of South Africans public property) (Kabata and Thaldar, 2023). However, apart from briefly referring to a “traditionally” held legal view regarding human biological *samples*, the ASSAf report does not present legal analysis on whether human genetic *data* satisfy the criteria for ownership in South African law.

Such an analysis has since been embarked on by Thaldar et al. (2022), showing that a human genetic data instance—i.e., the computer file containing the sequence data—is indeed susceptible of private ownership in South African law. This conclusion is important in the context of developing an open science policy, as private ownership rights in human genetic data can be a powerful tool to either facilitate or hinder greater access to such data. The *Draft National Open Science Policy*’s principle of “as open as possible, as closed as necessary” can only be sensibly applied if there is clarity regarding the parameters of legal rights in human genetic data.

### 2.3 Retain a clear differentiation between human and non-human genetic data

The *Draft National Open Science Policy* refers to the *Nagoya Protocol to the Convention on Biological Diversity* (Secretariat of the Convention on Biological Diversity, 2011) (the *Nagoya Protocol*) and states that: (a) the *Nagoya Protocol* deals with access to, and benefit sharing of, genetic resources; and (b) the *Nagoya Protocol* has “gained interest with the idea of extension to other genomic data” (Secretariat of the Convention on Biological Diversity, 2011). First, we discuss the exact legal scope of the *Nagoya Protocol*, and second, we suggest that the idea of extending the scope of the *Nagoya Protocol* is controversial and can only serve to detract from the positive vision of the *Draft National Open Science Policy*.

The *Convention on Biological Diversity* (Secretariat of the Convention on Biological Diversity, 1992) (the *Convention*) provides that each state has sovereign rights over its genetic resources, meaning that each state can decide how its genetic resources will be governed, including how rights will be vested in its genetic resources. This principle is often referred to as “genetic sovereignty”. But does this principle include *human* genetic data? The *Convention* defines “genetic resources” as “genetic material of actual or potential value” (Secretariat of the Convention on Biological Diversity, 1992); “genetic material”, in turn, is defined as “any material of plant, animal, microbial or other origin containing functional units of heredity” (Secretariat of the Convention on Biological Diversity, 1992). Since the word “animal” can be interpreted as including humans, the Conference of the Parties to the *Convention* (1995) clarified that the *Convention* does *not* apply to *human* genetic material. When adopting the *Nagoya Protocol*, the Conference of the Parties to the *Convention* (2010) again recorded that the *Nagoya Protocol* does *not* apply to *human* genetic material. Accordingly, the principle of genetic sovereignty is limited to non-human genetic material.

But, would it not be a good idea to lobby for an expansion of the *Convention* and the *Nagoya Protocol* to also apply to *human* genetic data? After all, human individuals that belong to the same ethnic group share certain genetic similarities. As such, if individual members of a certain ethnic group participate in a genetic research project, such individuals may be providing valuable genetic information, not only about themselves as individuals but about their entire ethnic group. Should such ethnic groups not be entitled to control access to—and benefit from—such genetic information? Moreover, from a national perspective, should the human genetic data of South Africans not be viewed as a natural resource, similar to water or gold, that should be managed by government as public property? This was indeed the position taken by the ASSAf report (ASSAf, 2018). However, we suggest that this position would be difficult to sustain in the South African context for the following legal and policy reasons.

#### 2.3.1 Reason 1

While non-human biological resources, such as indigenous fynbos flowers or butterflies, cannot decide for themselves whether to provide their genetic material for research and on what conditions, humans can. Underlying the rights entrenched in the South African Bill of Rights is “the constitutional celebration of the possibility of morally autonomous human beings independently able to form opinions and act on them” (British American Tobacco South Africa (Pty) Ltd v Minister of Health, 2012). Provided that individual autonomy is protected through informed consent, how will the South African government (or the leadership of a community) justify restricting individuals’ autonomy to donate their genetic data to research projects that they themselves deem worthy?

#### 2.3.2 Reason 2

Building further on this theme, it is important to note that non-human genetic data are *impersonal* in nature, while human genetic data are *personal* in nature (Shabani and Borry, 2018; Thaldar et al., 2019; Costello, 2022). This is an additional reason why the comparison of human genetic data with natural resources is misleading. While there are no personality rights in an indigenous fynbos flower or a butterfly—or in public property such as water or gold—persons have *personality rights* in their own genetic data. Personality rights are inseparably bound up with one’s personality, cannot exist independently of the human personality, and are incapable of being transferred (Kumalo v Cycle Lab (Pty) Ltd, 2011). Examples are the right to the integrity of a person, to respect a person’s name and reputation, the right to informational privacy generally (as codified in Protection of Personal Information Act 4 of 2013), and the right to control the use of one’s image. Accordingly, we suggest that the following is a more appropriate comparison: If persons belonging to, for example, ethnic group X, which is indigenous to South Africa, act as models in a commercial advertisement and are paid handsomely, should all persons identifying as belonging to ethnic group X share in benefits from the use of those individuals’ images? Moreover, would the South African government (or the leadership of ethnic group X) be justified in exercising control over the images of the individual persons who are members of ethnic group X and who voluntarily decided to participate in making the advertisement? When considering these questions, bear the following in mind: Model Y may look very similar to her biological sister, but this fact does not give Y’s sister any rights over the use of Y’s image. Similarly, patient Z who suffers from a heritable condition is at liberty to disclose the nature of her illness to whomever she pleases, despite the fact that such information will imply a certain genetic propensity towards the same heritable condition among her family members. There may be moral and cultural considerations applicable to Z’s decision, but legally she is perfectly entitled to disclose the nature of her illness to whomever she wishes.

#### 2.3.3 Reason 3

Health law in South Africa is based on the principle of altruism in research participation (Jordaan, 2016; Thaldar et al., 2021). The National Health Act 61 of 2003 provides (in section 60(4)) that research participants who donate tissue or blood samples may only be compensated for reasonable expenses incurred, and (in section 60(5)) that it is a criminal offence to offer such research participants financial or other reward (apart from reasonable expenses incurred) for their donation. Given the current state of genetic technology, genetic sequence data cannot be obtained directly from a research participant, but must be obtained from a human biological material sample. Accordingly, donating a human biological material sample is a *conditio sine qua non* for genetic research. This clearly restricts the kinds of benefit sharing that research participants in genetics research projects may lawfully receive in South Africa, as any type of benefit sharing that constitutes a “financial or other reward” for the research participant would be unlawful (and criminal) (Thaldar and Shozi, 2023). Importantly, the *Nagoya Protocol* is not self-executory, meaning that it only gains effect in South African law if, and to the extent that, it is incorporated into South African statute law. Accordingly, a hypothetical amendment to the *Nagoya Protocol* (the “idea of extension”) to include *human* genetic data would, on its own, not affect the legal reality in South Africa. Note that such an amendment is pure conjecture, as there is no indication that any party to the *Nagoya Protocol* intends to propose such an amendment.

#### 2.3.4 Reason 4

The final reason is the most fundamental in the present context. The idea of genetic sovereignty in the human context—where the state or a community exercises sovereign power over human genetic data—is philosophically opposed to open science. Genetic sovereignty, to have any meaning, will entail access barriers in the form of the state or a community deciding who can access human genetic data, and on what conditions. By contrast, open science entails access to research results—including human genetic data—*free of access barriers* (Steger and Hantho, 2019; Crow and Tananbaum, 2020). As recently argued by Kabata and Thaldar (2023), the idea of state sovereignty over human genomic (or genetic) data may seem superficially attractive, but has no actual utility to African states. Instead, the authors suggest a human-rights-based approach to the governance of human genetic data that focuses on everyone’s *right to science*, which is aligned with promoting open science (Kabata and Thaldar, 2023).

For these reasons, we suggest that the *Draft National Open Science Policy* should either remove the sentence about “the idea of extension” of the *Nagoya Protocol* to “other genomic data”, or add a clear disclaimer that such an idea is contrary to current South African law and contrary to the objective of the *Draft National Open Science Policy* to promote open science.

### 2.4 A brief comparison with the *Draft National Policy on Data and Cloud*

We now turn to the positive aspects of the *Draft National Open Science Policy* that we identified in the introduction above. Our analysis takes the form of a comparison of the *Draft National Open Science Policy* with another draft policy published for public comment by the South African government, namely, the *Draft National Policy on Data and Cloud* (Department of Communications and Digital Technologies, 2021) (see Table 1 below). It is relevant to note that these two draft policies were produced by two different government departments: the former by the Department of Science and Innovation; the latter by the Department of Communications and Digital Technologies. Many of the policy objectives of the *Draft National Policy on Data and Cloud* deserve support. These include: (a) encouraging universal access to broadband connectivity; (b) eliminating regulatory barriers and enabling competition in the data and cloud sector; (c) supporting the development of small, medium, and micro enterprises; and (d) promoting research, innovation, and technological developments in relation to the cloud. However, the *Draft National Policy on Data and Cloud* is premised on the ideological position that (drastically) greater state control of data is the best solution. For the reasons stated below, we are critical of this position.

**TABLE 1:** Differences between the *Draft National Open Science Policy* and the *Draft National Policy on Data and Cloud*.

*Draft National Open Science Policy*	*Draft National Policy on Data and Cloud*
Developed by the Department of Science and Innovation	Developed by the Department of Communications and Digital Technologies
Aims to facilitate free access to data through incremental, incentivized moves towards open science	Aims to facilitate free access to data through the nationalization of all data generated in South Africa
Operates within the existing intellectual property framework	Suggests the disruption of the intellectual property legal framework
Respects the right to private property	Suggests the disruption of private ownership of data

While there is an important overlap between the objectives of the *Draft National Open Science Policy* and the *Draft National Policy on Data and Cloud*, as both draft policies aim to facilitate South Africans’ free access to data, the two policies propose to accomplish this objective through radically different means: the *Draft National Open Science Policy* through incremental, incentivized moves towards open science, within the existing intellectual property legal framework and the right to private property; the *Draft National Policy on Data and Cloud*, on the other hand, through the nationalization of all data generated in South Africa, the disruption of the intellectual property legal framework, and government control of access to data.

At a level of principle, these two approaches are clearly ideologically incompatible. Which of these two approaches would be better aligned with the values of the open and democratic society that South Africa aspires to be? Without doubt, the *Draft National Open Science Policy*. When examining the concept of an “open society” in the South African Constitution, Justice Ackermann (in Ferreira v Levin, 1996) relied on Karl Popper’s *magnum opus*, *The Open Society and Its Enemies* (Popper, 1945). This judgment points to the political philosophical source of the concept of an “open society”, and opens the door to learn from this source (Jordaan, 2017). In *The Open Society and Its Enemies,* Popper (1945) famously proposed that policy-making (or social engineering) in an open society should be “piecemeal”, rather than “utopian”. Piecemeal social engineering denotes small-scale social “experiments” that can be modified or reversed based on results in the social “laboratory”. Utopian social engineering, on the other hand, denotes large-scale policy interventions that seek to modify human behavior to conform to policy ideas at any cost—i.e., without error-correcting mechanisms along the way (Popper, 1945). The *Draft National Policy on Data and Cloud*’s proposal to nationalize all data generated in South Africa is not only far-reaching, but contains no intermediate steps to provide for learning and adapting based on real-world effects. As such, it leans dangerously towards the kind of utopian social engineering that Popper warned against. Fortunately, the *Draft National Open Science Policy* does not fall into this trap.

Also at a practical level, the *Draft National Open Science Policy* offers a more realistic pathway to reaching the objective of facilitating South Africans’ free access to data. It respects the existing legal frameworks and individual rights, while envisioning a cultural change towards open science that will be championed by an official body, the Open Science Advisory Council, and incentivized along the way. By contrast, the *Draft National Policy on Data and Cloud* proposes to break down established legal frameworks and rights, and impose government control of data. It is human nature to respond positively to incentives, and to respond negatively to infractions on one’s reasonable expectations, such as the expectation that one’s individual rights will be respected. Furthermore, obvious questions can be raised regarding the efficiency of government control of data. Accordingly, if South Africans’ free access to data is the public policy objective, the *Draft National Open Science Policy* offers a much more attractive policy pathway to accomplish this objective.

## 3 Conclusion: towards open science in South Africa

The *Draft National Open Science Policy* is a milestone in South Africa’s journey towards a workable and effective national policy that promotes open science at all levels of scientific endeavor. However, there is room for improvement in the three areas that we have highlighted. In our view, the final national open science policy should seriously engage with constitutional law (the right to freedom of scientific research), property law (ownership), and international law (the *Nagoya Protocol*) aspects of human genetic data *qua* research output. This will ensure that the final policy is clearly anchored in the South African Constitution, and that the principle of “as open as possible, as closed as necessary” can be applied to human genetic data in a legally well informed and accountable way.

Note that the *Draft National Open Science Policy* was not made public by the South African Department of Science and Innovation. Instead, it was only disseminated via email to “stakeholders” within the South African academic community, who were given the opportunity to submit comments. It is anticipated that a subsequent version will, at some future stage, be published for public comment. To assist the reader, we include a summary of the *Draft National Open Science Policy* as Supplementary Material S1.

## Data Availability

The original contributions presented in the study are included in the article/Supplementary Material, further inquiries can be directed to the corresponding author.
