# Supplementary material for: Open science and human genetic data: recommendations on South Africa’s Draft National Open Science Policy
Source: Front Genet. 2023 Oct 2;14:1248747. doi: 10.3389/fgene.2023.1248747 (PMC10577200; doi:10.3389/fgene.2023.1248747)
Supplement: Supplementary file 1 [file DataSheet1.PDF]

Supplementary Material

Draft National Open Science Policy of South Africa: A Summary

1 Introduction and background

The concept of open science refers to the open, collaborative nature of scientific research that facilitates sharing and utilization of various research outputs. South Africa, in line with global trends, is navigating towards a more open and transparent research environment. The *Draft National Open Science Policy* aims to address existing inefficiencies, such as research and publication paywalls, which hinder broad access to research outputs.

2 Definitions and key concepts

The *Draft National Open Science Policy* establishes key definitions:

- **Open Science:** Transparent and accessible knowledge through shared resources whose outputs are publicly available.
- **Open Access:** Unrestricted access to peer-reviewed scholarly research.
- **Open Data:** Freely accessible, usable, and distributable data.
- **Open Source:** Making software code that has been used in the processing and analysis of research data accessible for re-use.
- **Publicly funded research outputs:** Outputs from research and development undertaken by individuals, teams or publicly funded research performing organizations using any funds allocated by a state funding agency.

The *Draft National Open Science Policy* establishes the boundaries between open science and intellectual property, respecting the delicate balance between sharing and protection.

3 Rationale and benefits

Open science promotes the democratization of knowledge. It:

- Speeds up scientific discoveries.
- Facilitates interdisciplinary research.
- Provides a platform for citizen science involvement.
- Boosts the transparency and reproducibility of research.
- Amplifies the socio-economic impact of research outputs.

- Strengthens research capabilities in previously disadvantaged institutions.

4 Open science in the national context

South Africa possesses a dynamic National System of Innovation (NSI). Open science aligns with the nation's drive towards inclusive development and innovation. Recognizing socio-economic inequalities, the *Draft National Open Science Policy* underscores the need for infrastructural development, especially internet access, as a cornerstone for the open science vision.

5 *Draft National Open Science Policy* vision and objectives

The adoption of a National Open Science Policy will facilitate equality of opportunity within South Africa's NSI. The *Draft National Open Science Policy* envisions a transformed research landscape where scientific knowledge, data, and resources are universally accessible and usable. Objectives include:

- Enhancing access to research outputs.
- Advancing inclusive economic development.
- Promoting collaboration and interdisciplinary research.
- Transforming the research culture to align with open science principles.

6 Scope of the *Draft National Open Science Policy*

The *Draft National Open Science Policy* covers scholarly publications, research data, software, educational resources, and other intellectual outputs from publicly funded research in South Africa. It also recognizes the diverse needs of various disciplines, ensuring flexibility in its application. When research is funded by the private sector or philanthropic funders, the *Draft National Open Science Policy* shall be applied on a best-effort basis which respects the confines of the contracts in place.

7 Principles of open science

The guiding principles shall be founded on the following core values:

- **Transparency and Accessibility:** Encouraging open dissemination of scientific knowledge.
- **Inclusivity:** Catering to all stakeholders, including researchers, educators, the public, and industry.
- **Collaboration:** Promoting shared research activities and outcomes.
- **Security:** Ensuring protection against potential misuse and breaches.
- **Quality:** Maintaining high research standards.
- **Ethics:** Upholding the principles of responsible research and publication.
- **Stewardship:** Ensuring that the shared data and research are well-managed and preserved.

The adoption and application of open science in South Africa shall be guided by the following principles:

- Publicly funded data and outputs must be findable, accessible (discoverable), interoperable, and reusable (FAIR) in terms of their dataset characteristics.
- The CARE principles (collective benefit, authority to control, responsibility, and ethics) complement and extend the more data-centric approach of the FAIR principles.
- Transparency, responsibility, user community, sustainability, and technology (TRUST) are essential components for assessing, developing, and sustaining the trustworthiness of data repositories, and thus supporting the scientist and other actors.
- Flexibility: Understanding that the application of open science requires a flexible, context-driven approach.
- Operational and financial sustainability.
- As open as possible, as closed as necessary.

8 Implementation: Resources, roles, responsibilities, and governance

8.1 Critical Success Factors identified to ensure an effective implementation of the *Draft National Open Science Policy*

- **Universal Access:** Bolstering internet coverage and online training.
- **Trusted Security:** Adopting high-standard security measures for data repositories.
- **Publishing Realities:** Recognizing global publishing norms.
- **Legislation:** Striking a balance between open science and intellectual property rights.
- **Sustainability:** Committing to long-term viability through feasibility studies and risk analysis.
- **Stakeholder Engagement:** Continuously involving the research community.
- **Change Management:** Iteratively testing and refining the Implementation Plan.
- **Global Compatibility:** Aligning with international open science platforms.
- **Consistent Inter-Departmental Policy:** The ability to implement consistent policy across departments.

8.2 Governance

- **Open Science Advisory Board (OSAB):** A governance body ensuring oversight.

8.3 Resources, stakeholders, and responsibilities

- Comprehensive implementation plan addressing stakeholders, resources, risks, and compliance.
- Involvement of government, research organizations, publishers, science academies, the private sector, non-governmental organizations (NGOs), civil society coalitions, and research funders.
- An incentive and compliance framework shall be adopted and tailored to the relevant parties.

9 Monitoring and evaluation

The **South African Open Science Observatory** will be pivotal in monitoring the *Draft National Open Science Policy's* effectiveness. This body will:

- Provide insights on open science trends in South Africa.
- Support the evaluation of open science across its multiple dimensions.
- Produce a dashboard with indicators, analytics, and visualizations.
- Remain under public oversight, ensuring transparency and efficiency.

Metrics will focus on open access, data, source, resources, methods, and review. There is an emphasis on continuous self-assessment, staying updated with international best practices, and adopting a hybrid model of evaluation.

10 Conclusion

Open science brings undeniable advantages, and as the global community embraces it, South Africa needs to align. The primary goal is to make publicly funded research beneficial for all citizens by ensuring responsible access, while also safeguarding privacy and consent. The transformation will lead to a broader reach of beneficiaries of science and pivot the nation towards a future of shared scientific discovery and innovation.

This comprehensive *Draft National Open Science Policy* is South Africa's roadmap to democratizing scientific knowledge. It sets out the vision, principles, and strategies to transform the nation's research landscape. By leveraging global trends, respecting intellectual property, and integrating best practices, South Africa aims to be at the forefront of the open science movement, ensuring that its scientific advancements benefit both its citizens and the world.
